# Evaluating the effect of prenatal interventions on maternal–foetal attachment: A systematic review and meta‐analysis

**DOI:** 10.1002/nop2.648

**Published:** 2020-10-06

**Authors:** Elieh Abasi, Afsaneh Keramat, Narjes Sadat Borghei, Shahrbanoo Goli, Maryam Farjamfar

**Affiliations:** ^1^ Student Research Committee School of Nursing and Midwifery Shahroud University of Medical Sciences Shahroud Iran; ^2^ Reproductive Studies and Women’s Health Research Center Shahroud University of Medical Sciences Shahroud Iran; ^3^ Counseling & Reproductive Health Research Center Midwifery Department Golestan University of Medical sciences Gorgan Iran; ^4^ Department of Epidemiology Shahroud University of Medical Sciences Shahroud Iran; ^5^ Clinical Research Development Unit Imam Hossein Hospital Shahroud University of Medical Sciences Shahroud Iran

**Keywords:** attachment, intervention, maternal**–**foetal attachment, meta‐analysis, systematic review

## Abstract

**Aim:**

This study aimed to evaluate the effect of prenatal interventions on maternal foetal attachment.

**Design:**

Systematic review and meta‐analysis.

**Methods:**

In this study, a comprehensive review was performed to find articles published from January 2000 ‐ December 2019 in the form of randomized and non‐randomized clinical trials. To this end, online databases including PubMed, Scopus, Google Scholar, ScienceDirect, Proquest, Ovid, CINAHL and JAMA were searched. Duplicate articles were also excluded using Endnote X7 Reference. The results were then analysed via RevMan 5.3 software.

**Results:**

The results showed that foetal movement counting did not seem to be effective in increasing MFA by itself. But, this intervention alongside other attachment behaviours such as touching the belly and talking to foetus could enhance MFA. Therefore, the best interventions to improve MFA might be combined ones implemented in the form of counselling and training sessions.

## 1 INTRODUCTION

Maternal**–**foetal attachment (MFA) is a term used to describe the relationship established between a pregnant woman and her foetus (Salisbury et al., 2003). This type of attachment essentially comes into being during pregnancy (Rubin, 1970, 1975). It should be noted that attachment theory was first set forth by Bowlby (1969) to point out the relationship between children and their caregivers, especially their mothers. In this respect, attachment was defined as the lasting emotional bond occurred between a mother and her child (Bowlby, 1969). The given theory has been thus far widely used at all stages of human development. The evolution of this theory before birth has been mainly started by nurses (Brandon et al., 2009).

As the founder of the theoretical structure of prenatal attachment, Rubin (1975) has also described four specific prenatal activities by mothers as (a) seeking security for mother and foetus, (b) ensuring that foetus is accepted by others, (c) establishing an internal bond with foetus and (d) self‐sacrificing. According to Rubin (1975), at the end of the second trimester, a pregnant woman becomes much more aware of the presence of an individual inside her body and feels more attached, so she has something very dear and important within herself, giving her a sense of pleasure and pride (Brandon et al., 2009; Rubin, 1975). Following Rubin, researchers such as Lamley, Leifer, Cranley, Muller and Condon have also worked in this field (Brandon et al., 2009). Accordingly, Muller defines attachment as exceptional relationship generated between a mother and her foetus (Müller, 1989). As stated by Cranley, MFA refers to the extent of engagement in behaviours denoting dependency and interaction with an unborn child (Cranley, 1981).

In the view of MFA, there is a gradual improvement in terms of time and how to express prenatal attachment. Researchers have further reflected on levels or severity of attachment at different stages of pregnancy (Doan & Zimerman, 2007). According to Leifer (1977), prenatal attachment evolves as a stage during pregnancy. Initially, a relatively low level of attachment is observed in the first trimester. Once foetal movement is felt, attachment increases and then it is intensified in the second and the third trimesters (Leifer, 1977). Obviously, prenatal attachment can range from initial high attachment to low attachment or no attachment during pregnancy due to individual differences (Mikulincer & Florian, 1999).

## 2 BACKGROUND

MFA can largely affect postpartum childcare and development. It can also predict potential health and well‐being of babies and infant health care costs (Alhusen et al., 2012). MFA may even affect dependency in postpartum mother–child relationships and maternal ability to care for a baby (Alhusen et al., 2013; Cannella, 2005; Shin et al., 2006).

Strong maternal bond has been correspondingly reported to be the basis for growth of pregnancy behaviours that include foetal care and protection (Cranley, 1981). In women reporting higher MFA in pregnancy, early childhood development is more favourable in their babies compared with those who report low attachment (Jeanne L Alhusen et al., 2013). Higher MFA can thus increase interactions with a foetus like eating properly, quitting alcohol use, having positive imaginations about a foetus, talking to a foetus, paying attention to foetal movements, and other interactive behaviours with a foetus that can lead to promotion of maternal**–**foetal health status (Salisbury et al., 2003; Yarcheski et al., 2009).

Research has likewise revealed that some prenatal interventions enhance attachment between a mother and an unborn child. When a mother suffers from prenatal stress, depression and discomfort, such interventions can help her demonstrate more maternal behaviours which can in turn improve her health status (Bowlby, 1969; Joanne, 2001; Moein, 2001). Various interventions have been thus far designed and implemented to facilitate attachment in many different ways, in particular intervention programmes using touch of pregnant belly (Carter‐Jessop, 1981), providing couples with child‐care knowledge (Bryan, 2000), giving information and using stress management training (Schachman et al., 2004), expressing feelings to the foetus through letter writing (Chang et al., 2004), and talking to the foetus and counting foetal movements (Salehi et al., 2017). Obtaining more information about foetal activity during prenatal care will likely aid pregnant women develop stronger emotional bonds with the foetus (Salehi et al., 2017).

According to a related research on attachment disorders, early detection and intervention are of utmost importance in this line. Further knowledge of the role of maternal**–**foetal attachment can be also regarded as a stimulus for development of prenatal interventions and prevent poor mother–child attachment bond. Along with the rising trend in empirical knowledge, methods of identifying mothers at risk of poor attachment and designing intervention programmes to prepare women for motherhood have been taken into serious consideration. Women with insecure attachment may respond to appropriate interventions and those unaware or indifferent to their attachment to the foetus may take advantage of training and motivation. Besides, the results of interventions highlight the significance of more knowledge about the concept of foetal attachment, asymmetrical nature of prenatal attachment, and factors accelerating its development and preventing its occurrence. Indeed, identifying families susceptible to insecure attachment can make it possible to intervene in this vicious cycle and set conditions for healthier attachment (Brandon et al., 2009). If low levels of maternal**–**foetal attachment are detected during pregnancy, appropriate interventions should be exercised to help pregnant women achieve a physically and mentally healthy pregnancy to promote maternal**–**foetal health (Alhusen, 2011).

Given the importance of these interventions and their effect on mother–child health, the present study was conducted to evaluate prenatal interventions. The purpose of this study was to briefly review the effect of interventions implemented during pregnancy on MFA to select appropriate ones to promote prenatal attachment.

## 3 DESIGN

Systematic review and meta‐analysis.

## 4 METHODS

### 4.1 Search strategy

This study was a systematic review and meta‐analysis of prenatal attachment‐related interventions. To this end, articles evaluating such interventions were reviewed and the data obtained were used in various sections of this study. The search strategy was formulated, and the relevant articles published from January 2000 until the end of December 2019 were explored in the databases of PubMed, Science Direct, Google Scholar, Proquest, Scopus, Ovid, CINAHL and JAMA. Duplicate articles were also excluded using Endnote X7 Reference. Also, a manual search of the reference lists of related studies was carried out to find more eligible publications. So, randomized and non‐randomized clinical articles (RCTs and non‐RCTs) in English were evaluated. To address the research question, statistical population, interventions, comparisons, outcomes and settings of the studies were considered (Picoc). Different keyword combinations were further employed to obtain maximum relevant articles (Table 1).

**Table 1:** Search items used

Maternal	Fetal	Attachment	Intervention
or	or	or	or
Prenatal	Fetus	Relation	Promotion
or	or	or	or
Mother	Baby	Bond	Clinical trial
or			or
Antenatal			Experimental study

### 4.2 Inclusion and exclusion criteria

Article selection criteria included studies published from 2000 to 2019, in English, on pregnant women, using interventions to promote attachment, measuring the total score of attachment in two groups and employing MFA questionnaires developed by Cranley, Muller and Condon. However, cross‐sectional, case–control, cohort and systematic review studies and those that had not reported the total score of attachment, publishing results unrelated to research objectives, with insufficient statistical data and low quality (by using CONSORT check list) were excluded.

### 4.3 Study selection and data extraction

**Figure 1:**
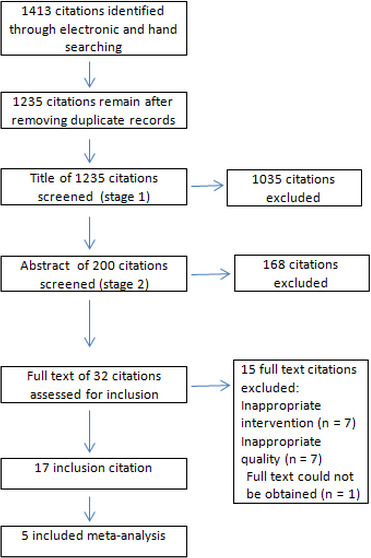
Flow diagram of study searching and selection process

Altogether, 1,413 articles were identified in the initial search in mentioned databases and other sources. Then, 1,235 articles among 1,413 articles were obtained considering time span and eliminating the duplicates. Having examined the titles and deleting cross‐sectional, cohort, case–control, systematic review and qualitative studies, approximately 200 articles remained. After reviewing these articles, 32 studies meeting the inclusion criteria were selected. Of these selected studies, in the case of two articles, we obtained the full text by contacting the authors via email. Reading the full‐text manuscripts of these articles and deleting some according to the Consolidated Standards of Reporting Trials (CONSORT), a total of 17 articles were ultimately analysed (Figure 1).

To increase the reliability of the study and to prevent bias in search, article selection, inclusion and exclusion criteria and evaluation of manuscripts were performed by two researchers independently. Difference of opinions was also resolved through discussions between the authors until a consensus was reached. Then, a checklist was used to obtain the required information for the study including the author's name, publication year, study setting, research design, sample size, instruments, interventions and results (Table 2). Moreover, statistical data including mean score of attachment before and after the interventions in two groups along with the titles of the studies were presented in Table 3, separately. Finally, RCTs using Cranley's Maternal**–**Fetal Attachment Scale (MFAS) with a five‐point Likert‐type scale and mean and standard deviation of attachment score reported before and after the intervention in both groups were included in the meta‐analysis.

**Table 2:** Characteristics of included studies

Authors (publication year) location	Study design, sample size, scale used	Type of Intervention	outcomes
Saastad et al.( 2011) Norway	RCT,951 women PAI (Mu¨ller)	Women in the intervention group received an information brochure, including instructions on how to use and interpret fetal movement charts, and were asked to count fetal movements daily from gestational week 28 by recording the time required to perceive 10 movements ‘‘fixed number’’(count‐to‐ten).	No difference was found between the groups in the scores on prenatal attachment.
GÜNEY and UÇAR (2019) Turkey	RCT,110 women MAAS	Training for fetal movement counting was provided to the experimental group. To increase the accuracy level of counting, pregnant women should start recording when they feel the first movement of the fetus and continue counting until they count 10 movements within 2 hr.	the maternal–fetal attachment score of the experimental group was found to be higher than that of the control group in the post‐test that was applied 4 weeks later.
Akbarzade et al. (2014) Iran	RCT,150 women MFAS (Cranley)	The interventions were performed during the 28th to 34th weeks of gestation. In doing so, the fathers were trained regarding the attachment skills through four 60–90‐min sessions held once a week. After the interventions, the fathers were followed up through telephone contacts and were asked to transfer their information to their wives. A reminder session was also held at the 38th week of gestation.	Training the fathers regarding the attachment behaviors and skills led to an increase in the maternal‐fetal attachment scores.
Delaram et al., (2018). Iran	RCT,208 Women MFAS(Cranley)	An information brochure was given to the women who were in the intervention group, and in this brochure they were trained how to count the fetal movements daily at morning from 28 weeks of gestation to 37 and record them on their chart. The intervention group began to count the fetal movements daily from 28 to 37 weeks of gestation. To ensure the fetal movement counting in the intervention group, one person of the research team telephoned the women every two weeks.	The counting of fetal movements by mother from 28 to 37 weeks of gestation did not affect the maternal‐fetal attachment in nulliparous women.
Salehi et al., (2017). Iran	RCT,52 women MFAS(Cranley)	Face to face education about counting and recording the daily fetal movement was provided by the researcher in the intervention group. They were asked to, for four weeks, lie down for half an hour after their breakfast every day on their left side to count and record the movements of the fetus. The subjects in the intervention group counted and recorded fetal movements from the 24th to the 28th week of the pregnancy in specific forms.	Education of fetal movement counting significantly increased maternal‐fetal attachment.
Chang et al., 2015. Taiwan (2015)	RCT,296 women MMFAS(Modified)	Participants in the experimental group were given the prerecorded CD and asked to listen to the music at least 30 min a day for 2 weeks, while they were at rest or at bedtime and on a self‐regulated basis, that they would feel more relaxed. The participants listened to the music in their preferred category, and were permitted to listen to the music either over speakers or through earphones.	No statistically significant differences in terms of perceived stress and maternal—fetal attachment were found between the post‐test results of the two groups.
Toosi et al., (2014) Iran	CT,84 primigravid pregnant woman, MFAS	Intervention group received educational program consisting of four 90‐min sessions over 4 weeks, one session per week on Saturdays in the third trimester of pregnancy. The prenatal education focused on physical fitness, emotional and mental health, health promotion and improving attachment behaviors. At the end of all sessions, the Benson Relaxation method was rehearsed.	Relaxation training reduces anxiety in pregnant women and improves maternal‐ attachment to the newborn.
Parsa et al., (2016) Iran	RCT, 110 women MFAS	Four weekly group sessions (consultation) during a month were conducted in case group. The sessions included training (pregnancy changes, nutrition, Symptoms of pregnancy risk, Focus on the fetus) and practical training.	Consultation of the MFA behavior improved MFA score.
Ekrami et al., (2020) Iran	RCT,80 women MFAS	The intervention group attended 1–3 individual and 6 group counseling sessions. The counseling sessions centered around such topics as expressing physiologic, anatomic and hormonal changes during pregnancy, understanding fetal development during various stages of pregnancy, The complications of unplanned pregnancy on the mother and fetus and continued postpartum complications, the way maternal‐fetal attachment is established, the significance of maternal‐fetal attachment during pregnancy and strategies for greater adaptation during pregnancy.	Counseling has a positive contribution to improving maternal‐fetal attachment in women with unplanned pregnancies.
Abasi et al., (2013) Iran	Interventional,83 women MFAS	They received education about MFA. In the first session, concepts such as attachment, MFA, benefits of attachment, and methods of performing attachment behavior were taught. These behaviors included counting fetus movements and recording them, positive imagination of fetus appearance, speaking to the fetus, imagining breastfeeding the baby, and touching the abdomen. Meanwhile, mothers were given forms to record these behaviors and were asked to complete them weekly. In the following sessions, how to practice these behaviors was discussed.	Training mothers on MFA behavior can enhance mother´s mental health and their attachment with the fetus.
Toosi et al., (2017) Iran	Semi‐experimental clinical trial,80 IVF women,MFAS	The mothers in the intervention group participated in four 90‐min educational classes (pregnancy changes, stages of IVF, physiology of fetal development, signs of anxiety, effects of anxiety on pregnancy, strategies for more adaptation with changes in pregnancy including appropriate nutrition, individual health, physical health,…) weekly. At the end of all the educational sessions, Benson's relaxation technique was performed.	Relaxation training was effective in reduction of anxiety and increase of maternal fetal attachment in the women who had used IVF to get pregnant.
Akbarzadeh et al., (2017) Iran	quasi‐experimental ,100 women, MFAS	Women in the intervention group received six weekly training sessions, which were held for 90 min based on the BASNEF model to present the educational materials. The titles of training sessions were: Promoting breastfeeding, correct postures for breastfeeding/importance of being in the mother's arms/proper weaning, maternal‐fetal attachment, formation of MFA/onset of attachment symptoms, father's role in MFA.	Training based on the BASNEF model could increase the maternal‐fetal attachment in nulliparous pregnant women.
Shin & Kim., (2011) Korea	quasi‐experimental,240women, MFAS	The experimental group received general prenatal care and single 30‐min session of music therapy during TVUS examination.	No significant difference were identified in stress and maternal‐fetal attachment.
Baghdari et al., (2015) Iran	quasi‐experimental 55 women, MFAS	In addition to the routine classes, the intervention group participated in four 60‐min sessions on adaptation to pregnancy. These sessions were facilitated by the researcher who was also a midwife. Moreover, the subjects in the experimental group were given an educational booklet and a CD concerning the outline of the education. The researcher called the mothers weekly to remind them to study the booklet and watch the CD.	The pregnancy adaptation training package increased the adaptation and maternal‐fetal attachment scores in pregnant women with a history of baby loss.
Azogh et al., (2018) Iran	quasi‐experimental 100women,MFAS	the intervention group received 4 sessions of cognitive behavioral training during 4 weeks. Content of the training program: Familiarity, unresolved grief, Psychological dimensions of pregnancy after stillbirth, Normal physiology of pregnancy, Stress management practices	Cognitive behavioral training improved maternal‐fetal attachment.
Kordi et al., (2016) Iran	clinical trial, 67 nulliparous women with unplanned pregnancy, MFAS	In the intervention group, one session of guided imagery on maternal role was performed in 34th week of pregnancy in groups of four to seven. Afterwards, guided imagery CDs were given to mothers to be performed at home twice a week for 2 weeks.	Guided imagery promoted maternal‐fetal attachment in women with unplanned pregnancy.
Jangjoo et al., (2019) Iran	RCT,71 women, with unplanned pregnancy, MFAS	Individuals from the intervention group participated in group counselling, which consisted of four sessions of 60 min during the 4 weeks in the third trimester of pregnancy (weeks 28–34).The objectives of sessions were: knowledge about the fertility system and attachment, fetus development stages and factors affecting it, Improving attachment, identification of signs of pregnancy risk and its treatment, Getting to know the stages of delivery and afterwards, getting to know the infant and its needs.	Intervention could significantly increase MFA scores and all its domains in pregnant women

**Table 3:** MFA before and after intervention

Study title	MFA score before intervention Mean(*SD*)	MFA score after intervention Mean(*SD*)	*p* value
Fetal Movement Counting—Effects on Maternal‐Fetal Attachment: A Multicenter Randomized Controlled Trial	EG: ‐ CG: ‐	EG: 59.54 (9.39) CG: 59.43 (9.35) *p* value: .747	‐ ‐
Effect of the fetal movement count on maternal–fetal attachment	EG: 70.78 ( 6.78) CG: 71.58 ( 7.54) *p* value: .536	EG: 78.41 (6.65) CG: 72.25 (7.16) *p* value: <.001	
The Effect of Fathers’ Training Regarding Attachment Skills on Maternal‐Fetal Attachments among Primigravida Women: A Randomized Controlled Trial	EG:55.98 ± 6.99 CG:‐ *p* value: .364	EG:61.90 ± 5.41 CG:‐ *p* value: .001	*p* value: <.001 *p* value: .660
The Effects of Fetal Movements Counting on Maternal‐Fetal Attachment: A Randomizsed Controlled Trial	EG: 90.23 ± 9.64 CG: 90.00 ± 10.04 *p* value: .866	EG:93.75 ± 7.59 CG:92.78 ± 9.90 *p* value: .433	‐ ‐
The Effect of Education of Fetal Movement Counting on Maternal‐Fetal Attachment in the Pregnant Women: a Randomized Controlled Clinical Trial	EG: 86.63 ± 11.62 CG: 87.48 ± 10.31 *p* value: >.05	EG: 96.30 ± 10.81 CG: 88.64 ± 10.31 *p* value: <.05	‐ ‐
The effects of music listening on psychosocial stress and maternal—fetal attachment during pregnancy	EG: 96.11 ± 19.9 CG: 92.04 ± 21.26 *p* value: >.05	EG: 100.96 ± 20.47 CG: 95.60 ± 22.83 *p* value: >.05	‐ ‐
The reduction of anxiety and improved maternal attachment to fetuses and neonates by relaxation training in primigravid women	EG: 60.1 ± 4.7 CG: 60.2 ± 4.5 *p* value: .897	EG: 63.6 ± 4.3 CG: 61.1 ± 5.1 *p* value: .017	*p* value: <.001 *p* value: .444
The effect of training and consulting on MFA in primigravid women: a Randomized Clinical Trial	EG: 88.60 ± 7.08 CG: 98.51 ± 11.44	EG: 102.82 ± 5.94 CG: 98.20 ± 11.55	*p* value: <.001 *p* value: .49
Effect of counseling on maternal‐fetal attachment in women with unplanned pregnancy: A randomized control trial	EG: 73.6 ± 8.9 CG:76.0 ± 9.4 *p* value: >.05	EG:96.6 ± 9.3 CG:76.5 ± 6.4 *p* value <.001	*p* value: <.001 *p* value: .14
The effect of maternal–fetal attachment education on maternal mental health	EG: 3.52 ± 0.5 CG: 3.45 ± 0.43 *p* value: .152	EG: 3.96 ± 0.38 CG: 3.42 ± 0.41 *p* value <.001	*p* value: <.001 *p* value: .374
The effect of relaxation on mother's anxiety and maternal Fetal attachment in primiparous IVF mothers	EG: 61.1 ± 4.4 CG: 61.0 ± 4.1 *p* value: .918	EG: 67.0 ± 2.9 CG: 62.0 ± 5.5 *p* value: <.001	‐ *p* value: <.001 *p* value: .231
Effect of the BASNEF model on maternal‐Fetal attachment in the pregnant women referring to the prenatal clinics affiliated to Shiraz University of Medical Sciences	EG:3.01 ± 21.20 CG:2.40 ± 21.38 *p* value: .74	EG: 4.63 ± 30.75 CG: 3.19 ± 22.20 *p* value: <.001	‐ ‐
Music therapy on anxiety stress and maternal‐fetal attachment in pregnant women during transvaginal ultrasound	EG:64.12 ± 11.53 CG:64.12 ± 11.53	EG:64.81 ± 11.51 CG:65.73 ± 13.08	*p* value: .659 *p* value: .659
The Effects of Pregnancy‐Adaptation Training on Maternal‐Fetal Attachment and Adaptation in Pregnant Women With a History of Baby Loss	EG: 66.25 ± 15.33 CG: 59.93 ± 22.13 *p* value: .28	EG: 75.75 ± 14.40 CG: 60.81 ± 15.95 *p* value: .003	*p* value: <.001 *p* value: .231
The effect of cognitive behavioral training on maternal‐fetal attachment in subsequent pregnancy following stillbirth	EG:83.50 ± 14.11 CG:78.10 ± 17.64 *p* value: .09	EG:92.36 ± 11.89 CG:80.90 ± 36.16 *p* value: <.001	*p* value: <.001 ‐
Effect of Guided Imagery on Maternal Fetal Attachment in Nulliparous Women with Unplanned Pregnancy	EG:88.40 ± 8.4 CG: 88.50 ± 10.7 *p* value: .966	EG: 94.26 ± 6.7 CG: 90.22 ± 9.5 *p* value: <.046	*p* value: <.001 *p* value: 0,005
Effect of counselling on maternal–foetal attachment in unwanted pregnancy: a randomized controlled trial	EG: 69.63 ± 10.26 CG: 65.03 ± 13.45 *p* value: .96	EG: 104.43 ± 22.51 CG: 64.79 ± 12.86 *p* value: <.001	*p* value: <.001 *p* value: .236

Abbreviations: CG, Control group; EG = Experimental group.

### 4.4 Quality appraisal

To shed light on the quality of the given articles, the CONSORT checklist 2010 (25item version) was employed. The CONSORT statement is primarily used to evaluate randomized, controlled trials (RCTs), but it has been extended to cover many other designs, like non‐inferiority and equivalence trials and reporting of harm‐related data (Boutron et al., 2008). The checklist, published in 1996 and revised in 2001, 2008 and 2010, includes a set of items to identify the powers and weaknesses of clinical trials for both pharmacologic and non‐pharmacologic treatments (Des Jarlais, 2004). So, studies accommodating at least 15 items were included. Table 4 presents a summary of the CONSORT evaluation of the included studies.

**Table 4:** Evaluation of include intervention studies using the 25‐item CONSORT checklist

	Title/Abstract	Background/Objective	Trial Design	Participants	Interventions	Outcomes	Sample size	Randomization	Allocation	Implementation	Blinding	Statistical methods	Participant flow	Recruitment	Baseline data	Numbers Analyzed	Outcomes and estimation	Ancillary analyses	Harms	Limitations	Generalizability	Interpretation	Registration	Protocol	Funding
Saastad et al. (2011)	■	■	◪	■	■	◪	■	■	■	◪	◪	■	■	◪	■	■	■	◪	◪	◪	◪	■	■	◪	□
Güney and Uçar (2019)	◪	■	■	■	■	◪	◪	■	■	■	◪	■	■	◪	■	■	◪	◪	◪	■	◪	■	□	□	□
Akbarzade et al.(2014)	■	■	■	■	■	◪	■	■	■	■	□	◪	■	◪	◪	■	◪	■	□	■	◪	■	■	◪	■
Delaram et al.(2018)	■	■	■	■	■	◪	□	◪	◪	■	□	■	■		■	■	◪	◪	□	■	◪	■	■	■	□
Salehi et al.(2017	■	■	■	■	■	■	◪	◪	◪	◪	◪	■	◪	◪	■	◪	◪	◪	□	□	◪	■	■	■	■
Chang et al. (2019)	◪	■	■	■	■	■	◪	■	■	■	□	■	■	◪	■	■	◪	◪	□	■	◪	■	□	□	■
Toosi et al.(2014)	◪	■	■	■	■	■	■	◪	◪	■	□	■	■	■	■	◪	◪	◪	□	■	◪	■	□	□	■
Parsa et al.(2016)	■	■	■	■	■	◪	□	■	◪	■	□	■	□	◪	■	◪	◪	◪	□	■	◪	■	■	□	□
Ekrami et al.(2020)	■	■	■	■	■	◪	■	■	■	■	◪	■	■	◪	■	■	◪	■	□	□	□	■	■	□	■
Abasi et al.(2013)	◪	■	◪	■	■	■	◪	◪	□	■	□	■	◪	■	■	■	◪	■	□	□	■	■	◪	□	■
Toosi et al.(2017	◪	■	■	■	■	■	◪	◪	◪	■	□	■	□	◪	■	■	◪	■	□	□	◪	■	□	□	■
Akbarzadeh et al.(2017)	◪	■	■	■	■	◪	■	□	□	■	□	■	◪	◪	◪	◪	◪	■	□	■	◪	■	□	□	■
Shin & Kim.(2011)	◪	■	■	■	■	■	■	□	□	■	□	■	■	◪	■	■	■	◪	□	■	◪	■	□	□	■
Baghdari et al.(2015)	◪	■	■	■	■	■	■	□	◪	■	□	■	■	◪	■	■	◪	■	□	■	□	■	□	□	■
Azogh et al.(2018)	◪	■	■	■	■	■	■	□	□	■	□	■	◪	◪	■	■	■	■	□	□	◪	■	□	□	■
Kordi et al.(2016)	◪	■	■	■	■	■	■	◪	◪	■	□	■	■	◪	■	◪	◪	■	□	■	◪	■	■	□	■
Jangjoo et al.(2019)	■	■	■	■	■	■	■	■	■	■	□	■	■	■	■	◪	■	■	□	■	◪	■	■	□	■

Note

■ present; ◪ present, with some limitations; □ not present

### 4.5 Synthesis of results

**Figure 2:**
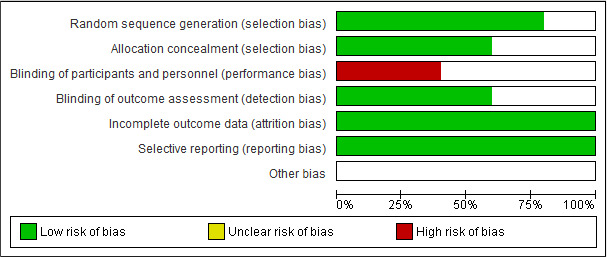
Risk of bias for all interventions

Statistical analysis was performed using RevMan 5.3 software (Review Manager Copenhagen: The Nordic Cochrane Centre). Five studies were also included in the meta‐analysis. The mean differences (MDs) with 95% confidence intervals (CIs) for foetal movement counting, counselling and total interventions were then estimated. What´s more, comparison of means was fulfilled using the random effects model. Study bias was correspondingly examined through RevMan 5.3 software and then reported in the form of low, high and unclear risks (Figure 2).

## 5 RESULTS

The eligible articles consisted of 9 RCTs and 8 non‐RCTs, respectively. Of the studies, 13 cases had been conducted in Iran and 4 other studies had been carried out in Norway, Turkey, Taiwan and Korea. Considering the need for interactions between participants and researchers in this type of interventions, performance bias in terms of blinding of the participant and the staff was reported as a high risk. Among 17 studies, 4 cases had examined the direct effect of foetal movement counting on MFA, where no impact had been observed in two studies in this respect (Delaram et al., 2018; Saastad et al., 2011) and two other articles had proven a positive effect (Güney & Uçar, 2019; Salehi et al., 2017).

Along with the above, in some studies, foetal movement counting had been regarded along with attachment behaviour. In other articles, interventions had been implemented in the form of training fathers about attachment skills (i.e. the impact of parental–foetal attachment on baby's psychological health status and parental anxiety, role of fathers in creating attachment, reducing maternal anxiety and success of postpartum breastfeeding), relaxation techniques (through relieving anxiety), counselling attachment behaviours (touching the belly, counting foetal movements, picturing foetal appearance, talking to foetus by mothers), pregnancy adaptation training (encouraging women to talk about feelings in their spouse and family about pregnancy, group discussions, examining mothers’ concerns about foetal health, spouse and family communication techniques and coping strategies for interpersonal problems), behavioural‐cognitive interventions (psychological aspects of pregnancy, normal physiology of pregnancy, stress management practices and problem‐solving patterns) and guided mental imagery (visualizing maternal role through mental imagery).

Two studies had also examined the effect of music therapy on MFA where listening to music in pregnancy had no impact on this type of attachment (Chang et al., 2015; Shin et al., 2006). Sample size also varied from 52–951 pregnant women in the studies. A total of 15 articles employed Cranley's MFAS, one study used Muller's Prenatal Attachment Inventory (PAI) and one case used Condon's Maternal Antenatal Attachment Scale (MAAS). In all studies, interventions were performed on pregnant women except one case where spouse training had been practiced as an intervention. None of the studies included postintervention follow‐ups.

**Figure 3:**
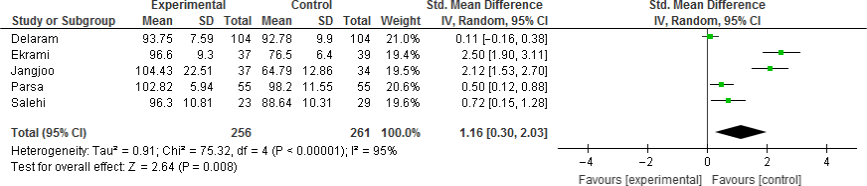
Forest plot for the meta‐analysis of all interventions

**Figure 4:**
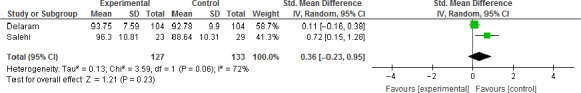
Forest plot for the meta‐analysis of foetal movement counting interventions

**Figure 5:**
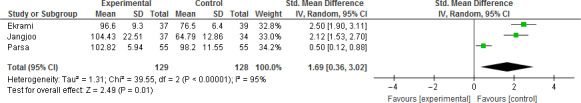
Forest plot for the meta‐analysis of counselling interventions

The results of the meta‐analysis confirmed that the overall effect of all interventions (i.e. foetal movement counting and counselling) was significant (MD = 1.16; 95% CI = 0.30–2.03; *p* value = .008) (Figure 3). But, no significant impact of two interventions on foetal movement counting was observed (MD = 0.36; 95% CI =−0.23–0.95 *p* value = .23) (Figure 4). Additionally, the effect of the three counselling interventions was significant (MD = 1.69; CI = 0.36–3.02; *p* value = .01) (Figure 5).

## 6 DISCUSSION

This study was a systematic review and meta‐analysis evaluating the effect of different interventions to enhance MFA. The results of the present meta‐analysis showed that various interventions had been, in general, effective in increasing this type of attachment (*p* value = .008). However, a meta‐analysis of two studies whose interventions had been foetal movement counting revealed that the given intervention was not effective in improving MFA (*p* value = .23). A review of three articles exercising counselling interventions had also demonstrated that such interventions had been effective in enriching MFA (*p* value = .01). The study by Delaram et al. (Delaram et al., 2018) on a larger sample size had further revealed no effect of foetal movement counting on this type of attachment. In their study, this intervention was performed for 10 weeks (at the gestational age of 28 to 37 weeks). In the investigation by Salehi et al, this intervention had been implemented with a smaller sample size for 4 weeks (at the gestational age of 24 to 28 weeks) (Salehi et al., 2017). Two other studies which were not included in the present meta‐analysis had also examined this intervention, where foetal movement counting enriched attachment in one study (Güney & Uçar, 2019), but no effect on MFA had been reported in the other one (Saastad et al., 2011). These discrepancies might be due to different statistical populations and time of interventions. Such contradictory results of studies in this domain suggested that further research was needed.

The meta‐analysis of three other studies in the form of counselling has correspondingly demonstrated that such interventions could increase attachment. Counselling sessions had usually lasted 4–6 sessions including presentation of specific issues associated with pregnancy such as physiological and hormonal changes during pregnancy, familiarity with attachment‐related behaviours, nutritional counselling, danger signs in pregnancy and focus on foetus along with practical exercises including touching the belly by pregnant mothers, guessing foetal position, counting foetal movement, picturing foetal appearance, talking to foetus and so on.

Other studies in this systematic review which were not included in the present meta‐analysis had also examined the impact of psychological interventions, apart from training and counselling aspects. The results of this section of the systematic review suggested that psychological interventions could facilitate MFA. In two studies, one case among nulliparous mothers and the other in mothers getting pregnant through in vitro fertilization (IVF), relaxation techniques had enhanced MFA via reducing anxiety (Toosi et al., 2017; Monire Toosi et al., 2014). Relaxation had thus affected different aspects of stress (Deckro et al., 2002; Öst & Breitholtz, 2000), and lower levels of maternal stress had drawn their attention towards the foetus.

In the study by Azogh et al., cognitive‐behavioural training had been performed in mothers with poststillbirth pregnancy which had improved attachment (Azogh et al., 2018). Cognitive‐behavioural training had been accordingly designed to correct wrong attitudes and beliefs and trust in current pregnancy. The psychological training provided in this method had enabled mothers to feel more attached to their foetus by communicating based on trust and empathy and encouraging them to review and express their previous pregnancy memories, touching the belly and making a relationship with the foetus, expressing emotions and positive and negative thoughts and accepting emotions by trainers and other group members (Azogh et al., 2018).

Kordi et al., 2016 in a study had examined the effect of guided imagery on MFA in unwanted pregnancy. Guided imagery had thus influenced individuals’ physical and psychological states and behaviours, promoted health status, relieved stress and consequently enhanced positive emotions (Zahourek, 1988). This intervention had also moderated maternal stress and anxiety‐related symptoms in pregnant women (Jallo et al., 2014). In this way, guided imagery during pregnancy could improve mothers’ feelings about their foetus and help them perform their roles (Kordi et al., 2016).

Two studies had also evaluated the impact of music therapy on MFA, suggesting that listening to music during pregnancy had no effect on attachment. In the study by Shin et al., listening to music for 30 min had occurred during vaginal sonography and at the gestational age range of 11–14 weeks (Shin et al., 2006). At the early stages of pregnancy, women would become less aware of foetus due to lack of foetal movement. So, music therapy could have little effect on MFA. In the study by Chang et al. (Chang et al., 2015), music intervention for 2 weeks in pregnant women over 17 weeks had not affected MFA.

## 7 CONCLUSION

The results of the present systematic review and meta‐analysis showed that foetal movement counting alone did not seem to be effective in increasing MFA. But, this intervention along with other attachment behaviours such as touching the belly, talking to foetus and the like could enhance this type of attachment. So, the best interventions to enrich MFA could be probably combined ones in the form of counselling and training programmes. Prenatal training could further have a positive effect on mothers’ awareness and self‐esteem (Hillier & Slade, 1989). Counselling and training focusing on strengthening attachment could thus increase it. Behaviours reinforced by MFA such as touching the belly, counting foetal movement, picturing foetal appearance, talking to foetus by mothers, training parents about foetal characteristics, focusing on attachment role and coping with pregnancy could bring more maternal attention to foetus. More awareness of foetus through these experiences might consequently support the existence of a foetus as an individual inside a mother (Laxton‐Kane & Slade, 2002). Therefore, mothers could become more attached to their foetus and attachment enhancement could be also effective in promoting mother‐foetus health status.

This systematic and meta‐analysis review study provides a summary of the available evidence about maternal**–**foetal attachment interventions and can be a pattern for selecting the best intervention to promote MFA for the wider global clinical community. It seems that these simple, inexpensive and effective interventions can be proposed as effective interventions to enhance attachment. Results of the current study could help the pregnancy care specialists in creating more appropriate evidence‐based decisions during pregnancy.

In this respect, midwives are often recognized as the first agents between mothers and healthcare teams, who are in frequent contacts with patients. Additionally, they are in the position of assessing and diagnosing women subjected to poor attachment and low emotional health with the purpose of early interventions.

It should be noted that this study also had some limitations. Most studies have been conducted in Asian countries, most of which are related to Iran. Many studies on attachment interventions in Iran in the period from 2000 to 2019 have been conducted that have met our criteria. Besides, some unpublished and lost information can lead to misinterpretation of the results.

## CONFLICT OF INTEREST

Authors declare that they have no conflict of interests.

## AUTHOR CONTRIBUTIONS

All authors: Contribution and met the four criteria for authorship recommended by the international committee of medical journal editors. All authors: Reviewing and approval.

## ETHICAL APPROVAL

The current review study was approved by the Ethical Committee of Shahroud University of Medical Sciences (Ethics No. IR.SHMU.REC.1398.019).

## Data Availability

The data used and analysed in this study are available from the corresponding author for reasonable requests.
